# Supplementary material for: Partners at Care Transitions (PACT)—exploring older peoples’ experiences of transitioning from hospital to home in the UK: protocol for an observation and interview study of older people and their families to understand patient experience and involvement in care at transitions
Source: BMJ Open. 2017 Dec 1;7(11):e018054. doi: 10.1136/bmjopen-2017-018054 (PMC5719264; doi:10.1136/bmjopen-2017-018054)
Supplement: Supplementary file 1 [file bmjopen-2017-018054supp001.pdf]

PACT WP1 Interview Guide

Section1: Getting to know people (ADMISSION)

Find out about their life e.g. where they live, who they live with, what job they used to do, do they have any children etc.

Section 2: Being admitted to hospital (ADMISSION)

a) Why have they been admitted to hospital? How did they get here?

   If they mention a condition, is it their main/only health concern? If not, what is?

   Probe for: **causative factors,** *expected duration of problems*, **expectations of treatments etc**, *impact on life*, **(if the problem preceded this hospital admission) what/who helps them to cope with/manage daily life?**

b) Could anything have avoided them coming into hospital?

c) Before they came into hospital, what contact did they have with health and/or social care professionals? Is this normal for them?

d) How do they feel about being in hospital?

e) What makes hospital care 'good'? What would make it better?

f) What do they think will happen next? What information have they been given? Do they feel they feel they have had had enough information?

   Probe for: **patient's understanding of why things are happening,** *how they know what is happening.*

g) How do they feel about going home?

Section 3: Questions about involvement (ADMISSION & POST-DISCHARGE & FINAL INTERVIEW)

h) How involved have they been in discussions about them and their treatment and care? How do they feel about this? Probe for: *choice*, **decision-making,** *information (given and received)*, **consultation about discharge process?**

i) How involved have they been able to be in their self-care whilst in hospital? E.g. normal daily activities?
j) What things are they able or would like to be able to do for themselves?
k) What would they like to be done for them?
l) Who else is involved in their care (e.g. family members)?
m) Do they have any questions about their condition, treatment, or care? If so, have they asked anyone these questions?

Section 4: Health and social issues (ADMISSION & POST-DISCHARGE – all interactions)

How are they managing their medication (getting them, taking them, understand them)? Have they fallen/problems with mobility? Any problems with equipment (e.g. catheters or adaptive equipment)? Any wound problems? Pressure ulcers? Appetite and thirst? Sleep? Energy levels? Pain (if so, well-managed)? How are they managing with normal daily activities (e.g. washing, dressing, going to the toilet, getting around, shopping, seeing friends and family)? Company? Any issues with appointments (making them, keeping them, or travelling to them)?

Section 5: Perceptions of risks & concerns at the moment (ADMISSION)

n) QUESTION: 'How safe and cared for do you feel at the moment?' (Probe: why/why not)
o) Has there been anything that has concerned them about the care they've had since being in hospital?
p) Is there anything in your life that is worrying or concerning them at the moment?
q) Have they shared their concerns with anyone? (Prompt for details e.g. who, how did they do it)
r) Do they have any ideas about what could make them feel… better/less worried/more comfortable/more confident (use patient's own words if appropriate)?
s) What things do they wish that staff knew and understood about them and their life?

Section 6: Perceptions of risks & concerns about the future (ADMISSION)

t) What issues do they think they could face when they leave hospital to go home?

u) Have they spoken to anybody about these things? If so, who?

v) Do they have any ideas about what things could be done so that [issues raised] don't happen/are avoided?

w) QUESTION: What do you think might be expected of you when you get home?

Section 7: Gaining an update (ALL SUBSEQUENT INTERACTIONS)

x) How are they at the moment?

y) QUESTION: Can you tell me what has happened to you [today…yesterday…etc/since I saw you last/since you came into hospital]? (Probe for their understandings about why these things have happened)

z) What have people done to help them feel supported and cared for, recently?

aa) How involved have they been in their care? How do they feel about this?

Section 8: Being at home (POST-DISCHARGE)

bb) QUESTION: How do you feel about being at home?

cc) Can you tell me what's happened since the last time I spoke to you (give day/date/location) if possible?

dd) What do you think and feel about [what has happened to you]?

ee) Who has been providing support or help since you came home?

ff) Do they feel that life is back to normal now? What have they been doing to make life as normal as possible? (Prompt for motives)

gg) What makes it easier to come home after being in hospital? What could stop them going into hospital?

hh) If they needed help with anything, what would they do/who would they ask?

<u>Section 9: Summarising (FINAL INTERVIEW)</u>

ii) Thinking about being in hospital, what was good? What could have been better?

jj) Thinking about the discharge, what was good? What could have been better?

kk) Did they feel ready to be discharged?

ll) Thinking about any treatment or care they have had since being at home, what has been good? What could be better?

mm) Looking back, is there anything that anyone or anything the hospital did that made it easier for them to come home?

nn) Is there anything that anyone has done for them/they’ve done for themselves that has helped them (get back to ‘normal’/avoid going back into hospital/stay at home)?

Section 10: Readmission (READMISSION)

oo) Why have they been readmitted to hospital?

pp) How did they come to be in hospital? (Prompt: did someone refer them? Transport to hospital?)

qq) Before they came into hospital, what contact did they have with HSCPs? Is that normal for them?

rr) How do they feel about being back in hospital?

ss) What do they think is going to happen next?

tt) QUESTION: Do you think anything could have avoided you having to come back to hospital?
